# Distributionally robust optimization for fire station location under uncertainties

**DOI:** 10.1038/s41598-022-08887-6

**Published:** 2022-03-30

**Authors:** Jinke Ming, Jean-Philippe P. Richard, Rongshui Qin, Jiping Zhu

**Affiliations:** 1 State Key Laboratory of Fire Science, University of Science and Technology of China, Hefei, 230026 China; 2 Department of Industrial and Systems Engineering, University of Minnesota, Minneapolis, 55455 USA

**Keywords:** Mathematics and computing, Applied mathematics

## Abstract

Emergency fire service (EFS) systems provide rescue operations for emergencies and accidents. If properly designed, they can decrease property loss and mortality. This paper proposes a distributionally robust model (DRM) for optimizing the location of fire stations, the number of fire trucks, and demand assignment for long term planning in an EFS system. This is achieved by minimizing the worst-case expected total cost, including fire station construction cost, purchase cost for fire trucks, transportation cost, and penalty cost for not providing adequate service. The ambiguity in demands and travel durations distributions are captured through moment information and mean absolute deviation. A cutting plane method is used to solve the problem. Due to fact that it is computationally intensive for larger problems, two approximate methods are introduced; one that uses linear decision rules (LDRs), and another that adopts three-point approximations of the distributions. The results show that the heuristic method is especially useful for solving large instances of DRM. Extensive numerical experiments are conducted to analyze the model’s performance with respect to different parameters. Finally, data obtained from Hefei (China) demonstrates the practical applicability and value of the model in designing an EFS system in a large metropolitan setting.

## Introduction

According to the International Association of Fire and Rescue Services^1^, increasing urbanization and industrialization leads to higher fire risks. For instance, the Ministry of Emergency Management of the People’s Republic of China reports that 1987 people died and 2225 were injured in fire accidents in 2021. Most fire accidents in China can be traced back either to electrical malfunctions or to lack of supervision of fires used for heating or cooking in family homes. Essential emergency fire service (EFS) systems provide a guardrail against the risks posed by such fires. Efficient EFS systems should make it possible to rapidly respond to calls, to provide timely rescue service, and to save lives^2,3^. Many studies have been conducted to improve the design of EFS systems. Boye et al.^4^ used Geographic Information System techniques to select appropriate sites for placing fire hydrants. When applied to a case study in Tarkwa, Ghana, results revealed that, with a travel speed of 80 km/h, the international standard of 4 minutes response time could be met for the entire area under study. Kiran et al.^5^ employed an enhanced floating catchment method to measure the spatial accessibility to fire stations. The identification of sites with lower level of spatial accessibility to fire services can then be used to make strategic investment decisions in fire resources. Similarly, Xia et al.^6^ proposed a two-step floating catchment area model to measure urban fire service access.

Fire stations are an important part of EFS systems. Their locations crucially influence the ability of an EFS system to respond to fires. Therefore, the network of fire stations supporting a given community should be designed so as to guarantee access to service and the resources for fighting fire incidents should be allocated to enable efficient response. However, trade-offs between efficiency and equity and between service performance and cost, as well as inherent characteristics such as travel time uncertainties, make this problem difficult. As a result, the EFS location and sizing problem has attracted considerable research attention as evidenced by numerous papers^7–15^.

In this paper, an EFS location model is introduced for planning the location of fire stations to be used in emergencies. The model considers fire events and travel times between candidate fire station locations and fires to be uncertain. It considers travel times above a given standard response time to be undesirable. It seeks to determine which fire stations to open, what demand areas to assign them to, and the number of trucks to equip them with so as to provide high worst-case service at lowest possible long-term operational cost.

Based on historical fire data from 2010, Fig. 1 presents statistics for travel durations to six types of places (Residence, Business, Dormitory, Restaurant, Warehouse, and Plant) in Hefei, China. As shown, variability exists in fire rescue durations, which leads to difficulties in accurately predicting travel durations in advance. There exist several types of methods for dealing with such fuzzy situations. Examples can be found in the literature^16–19^. Many studies on fire station siting problems require the distribution of uncertain variables to be known exactly^20–25^ as they rely on capturing the uncertainties in travel times through stochastic programming models or chance constraints. When distributions have large or infinite support, the sample average approximation technique can be used to produce approximate models that rely on discrete distributions, models for which a deterministic equivalent can be built. In this case, the decision-maker is risk-neutral and evaluates overtime via sample average. The approximation accuracy improves as the size of the sample increases while computation becomes more cumbersome. However, in many situations, high-quality data is limited and distributions for the data can be difficult to estimate.

**Figure 1:**
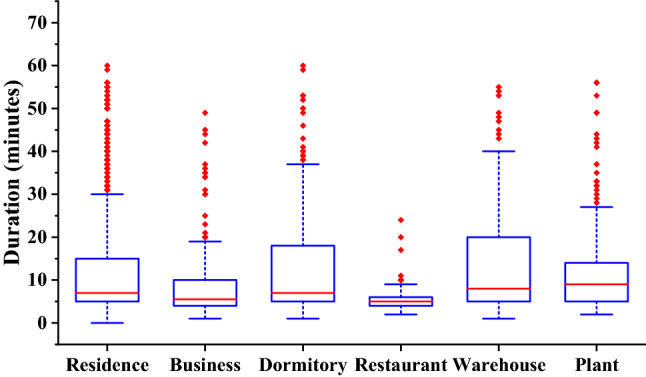
Fire response time variability among six kinds of fire events based on 1-year data.

Instead, to handle inherent uncertainties in EFS systems, this paper introduces a DRO model that does not require distributions of uncertain parameters to be known exactly. Distributionally robust optimization (DRO) is an emerging modeling approach that optimizes against the worst case of family of distributions; the collection of random variables in this family is defined through an ambiguity set that may restrict distribution properties such as moments and variances. This paradigm has been successfully applied to many areas, such as portfolio optimization^26^, power systems^27,28^, process scheduling^29,30^, and emergency service^31–33^. Its theoretical underpinnings can be found in^34,35^ among others. Compared with more traditional approaches considering stochastic factors, such as robust optimization and scenario-based stochastic programming, the DRO approach has the following advantages: (1) Users do not have to develop complex probability distributions for the stochastic elements of their models; and (2) DRO can utilize the data on hand to limit the family of random variables considered, which alleviates the over-conservatism of traditional robust optimization (RO) approaches.

Despite these advantages, to the best of our knowledge, there exist few studies which use the DRO paradigm to solve emergency facility siting problems. Liu et al.^31^ developed a two-stage risk-averse DRM for solving the emergency medical service station location and sizing problem. Yang et al.^36^ pointed out that pre-positioning emergency supplies is a crucial problem and proposed a DRM for the multi-period dynamic pre-positioning of emergency supplies with a static pre-disaster phase and a dynamic post-disaster phase.

In this paper, we propose a DRM for the siting and sizing of fire department locations in large urban areas. The contributions of this work are summarized next.

1. This work represents the first time that the EFS station location and sizing problem is formulated as a risk-averse distributionally robust model (DRM), simultaneously considering uncertainty in demand and in rescue time.
2. We show how the DRM we introduce for the problem can be transformed into an equivalent Mixed Integer Linear Programming (MILP) model through the use of duality theory. The number of constraints in this formulation is however exponential in the number of fire stations and demand sites. We introduce three solution methods for this model, one exact and two approximate and test their performance (both in terms of solution quality and solution times) on practical instances of the problem.
3. Extensive numerical experiments show that the heuristic method provides high quality solution at a fraction of the solution time required by the other two methods, especially for large instances of the problem.
4. We construct a large-scale practical dataset based on historical data collected from 2002 to 2011 for the city of Hefei, China. The heuristic method is used to solve this instance and to demonstrate its benefits. Valuable managerial insights are derived.

The remainder of the paper is organized as follows. First, the problem is described and a formulation is presented. Second, practical data is obtained and processed to create the inputs of the model. This includes, for instance, information about fire events, about their locations, and about travel time distributions between locations in Hefei. Third, numerical experiments are conducted to analyze the DRM’s performance with respect to different parameters and to validate its applicability on a large-scale practical application. Last, conclusions and directions for future work are discussed.

## Model formulation

This section introduces the concept of ambiguity set and presents the distributionally robust model (DRM) that will be studied in this paper for optimizing the location of fire stations. Three solution methods are then described to solve DRM.

### The concept of ambiguity set

In the context of stochastic programming (SP), it is common to seek a risk-neutral decision $x \in X$ by minimizing the expected value of random functions $\psi$(**x**, **T**) and $\phi$(**x**, **D**):

1 $$\begin{aligned} \min _ {x \in X} \left( \mathbb {E}_F\psi (\mathbf{x} , \mathbf{T} ) + \mathbb {E}_G\phi ( \mathbf{x} , \mathbf{D} )\right) , \end{aligned}$$

where $\mathbf{T} = [T_1, T_2, \dots , T_{M1}]$ and $\mathbf{D} = [D_1, D_2, \dots , D_{M2}]$ denote an M1-dimensional random vector and an M2-dimensional random vector respectively, showing uncertainties such as demands and travel times, and where **x** represents decision variables. However, in the absence of exact knowledge about the distribution of uncertainties, solving Eq. (1) can become difficult. Instead of considering explicit distributions for *F* and *G* in Eq. (1), the objective function of DRO intends to optimize the worst-case expectation of $\psi$(**x**, **T**) (resp., $\phi$(**x**, **D**)) among all possible distributions *F* (resp., *G*) in the ambiguity set ${\mathcal {F}}$ (resp., ${\mathcal {G}}$). This leads to the problem

2 $$\begin{aligned} \min _ {x \in X} \left( \sup _ {F \in {\mathcal {F}}}\mathbb {E}_F\psi (\mathbf{x} , \mathbf{T} ) + \sup _ {G \in {\mathcal {G}}}\mathbb {E}_G\phi ( \mathbf{x} , \mathbf{D} ) \right) . \end{aligned}$$

The ambiguity set includes a family of probability distributions that satisfy common statistical properties that can be estimated from historical data. General formulations for the ambiguity sets ${\mathcal {F}}$ and ${\mathcal {G}}$ are shown below:

3 $$\begin{aligned}&{\mathcal {F}} = \left\{ F \in {\mathcal {P}}_0(\mathbb {R}^{|I|*|J|}) \left| \begin{array}{ll} \mathbb {P}\{ T \in \mathbb {E}_1 \} = 1 &{} \\ \mathbb {E}_F \{ {\varvec{ {T}}} \} = \varvec{\mu }_\mathbf{T} &{}\\ \mathbb {E}_F \{ |{\varvec{{T}}} - \mu _\mathbf{T} | \} \le \varvec{\sigma }_\mathbf{T} &{} \end{array} \right. \right\} \end{aligned}$$

4 $$\begin{aligned}&{\mathcal {G}} = \left\{ G \in {\mathcal {P}}_0(\mathbb {R}^{|I|}) \left| \begin{array}{lr} \mathbb {P}\{ D \in \mathbb {E}_2 \} = 1 &{} \\ \mathbb {E}_F \{ {\varvec{{D}}} \} = \varvec{\mu }_\mathbf{D} &{}\\ \mathbb {E}_F \{ |{\varvec{{D}}} - \mu _\mathbf{D} | \} \le \varvec{\sigma }_\mathbf{D} &{} \end{array} \right. \right\} \end{aligned}$$

The first constraint in Eq. (3) or Eq. (4) ensures that ${\mathcal {F}}$ or ${\mathcal {G}}$ only contain valid distributions supported over their support sets $\mathbb {E}_1$ or $\mathbb {E}_2$. The remaining constraints in Eq. (3) or Eq. (4) characterize moment information and the information on mean absolute deviations of uncertainties. The support sets $\mathbb {E}_1$ and $\mathbb {E}_2$ are given as follows:

5 $$\begin{aligned} \mathbb {E}_1 = \left\{ {\varvec{{T}}}|\underline{{\varvec{{T}}}} \le {\varvec{{T}}} \le \overline{{\varvec{{T}}}} \right\} ,\,\, \mathbb {E}_2 = \left\{ {\varvec{{D}}}|\underline{{\varvec{{D}}}} \le {\varvec{{D}}} \le \overline{{\varvec{{D}}}} \right\} . \end{aligned}$$

By introducing two epigraphical random vectors ***v*** and ***u*** for the terms $|\mathbf{T}\mathbf - \varvec{\mu }_ {\varvec{{T}}}|$ and $|\mathbf{D}\mathbf - \varvec{\mu }_ {\varvec{{D}}}|$, the ambiguity sets and support sets Eqs. (3)–(5) can be rewritten as follows^32,37^:

6 $$\begin{aligned}&\mathcal {\overline{F}} = \left\{ F \in {\mathcal {P}}_0(\mathbb {R}^{|I|*|J|}) \left| \begin{array}{ll} \mathbb {P}\{ T \in \mathbb {E}_1 \} = 1 &{} \\ \mathbb {E}_F \{ {\varvec{ {T}}} \} = \varvec{\mu }_\mathbf{T} &{}\\ \mathbb {E}_F \{ {\varvec{{v}}} \} \le \varvec{\sigma }_\mathbf{T} &{} \end{array} \right. \right\} , \end{aligned}$$

7 $$\begin{aligned}&\mathcal {\overline{G}} = \left\{ G \in {\mathcal {P}}_0(\mathbb {R}^{|I|}) \left| \begin{array}{ll} \mathbb {P}\{ D \in \mathbb {E}_2 \} = 1 &{} \\ \mathbb {E}_F \{ {\varvec{{D}}} \} = \varvec{\mu }_\mathbf{D} &{}\\ \mathbb {E}_F \{ {\varvec{{u}}} \} \le \varvec{\sigma }_\mathbf{D} &{} \end{array} \right. \right\} , \end{aligned}$$

where the domain of uncertainties is extended from Eq. (5) to the two lifted support sets $\overline{\mathbb {E}}_1$ and $\overline{\mathbb {E}}_2$:

8 $$\begin{aligned}&\overline{\mathbb {E}}_1 = \left\{ ({\varvec{{T}}}, {\varvec{{v}}}) \left| \begin{array}{ll} \underline{{\varvec{{T}}}} \le {\varvec{{T}}} \le \overline{{\varvec{{T}}}} &{} \\ |\mathbf{T}\mathbf - \varvec{\mu }_ {\varvec{{T}}}| \le {\varvec{{v}}} &{} \end{array} \right. \right\} . \end{aligned}$$

9 $$\begin{aligned}&\overline{\mathbb {E}}_2 = \left\{ ({\varvec{{D}}}, {\varvec{{u}}}) \left| \begin{array}{ll} \underline{{\varvec{{D}}}} \le {\varvec{{D}}} \le \overline{{\varvec{{D}}}} &{} \\ |\mathbf{D}\mathbf - \varvec{\mu }_ {\varvec{{D}}}| \le {\varvec{{u}}} &{} \end{array} \right. \right\} , \end{aligned}$$

### Problem formulation under uncertainties

We study an EFS system that is composed of multiple fire stations and demand sites. Relief supplies such as fire trucks are stored in each fire station to satisfy uncertain demands from demand sites. Travel time from fire stations to demand points is uncertain. Therefore, two random variables are considered: the monthly demand ($D_i$) for fire trucks occurring at demand site *i* and the travel time ($T_{ij}$) from station *j* to site *i*. The former represents the number of calls for fire trucks in 30 days. The reason for setting a monthly period for demands is that it is in line with the way kernel density surface estimation is typically used for discrete fire records. The latter stands for fire response time. This research aims to find optimal fire station locations and to assign them a proper number of fire trucks so as to minimize the cost of the system, which includes construction cost, transportation cost, fire trucks purchase cost, and penalties associated with mismatch between supply and demand.

The parameters and decision variables used throughout the paper are summarized in Tables 1 and 2, respectively.

**Table 1:** Parameters used throughout the paper.

*I*	Set of demand points
*J*	Set of potential facility sites
$\theta$	Unit transportation cost
$\eta$	Per-unit cost for overtime
$\beta$	Per-unit cost for unsatisfied demands
$D_i$	Random variable that represents the demand weight at point *i*
$T_{i j}$	Random variable that represents the travel time from site *j* to point *i*
$T_0$	Standard fire response time
*P*	Maximum number of vehicles that can be placed in one fire station
*C*	Number of fires one fire truck can respond to during a month
$M_s$	Construction cost of one fire station
$M_v$	Purchase cost of one fire vehicle

**Table 2:** Decision variables.

$N_j$	Number of vehicles placed at site *j*
$Z_j$	Binary decision of locating a fire station at site *j* or not
$X_{i j}$	Number of fire trucks dispatched from fire station *j* to demand point *i* during 1 month

We use boldface letters to represent vectors or matrices. Observe that $\mathbf{T} \in \mathbb {R}^{(|I| * |J|)*1}$ and $\mathbf{X} \in \mathbb {R}^{(|I| * |J|)*1}$, respectively. The fire station location-allocation problem is formulated as a risk-averse DRM under two kinds of uncertainties, as follows:

10 $$\begin{aligned} &w^* = \min \sum _{j \in J} (Ms\cdot Z_j + Mv \cdot N_j) + \sup _{F \in \overline{{\mathcal {F}}}} \mathbb {E}_F[\theta \sum _{i \in I}\sum _{j \in J} T_{ij}X_{ij} + \eta \sum _{i \in I}\sum _{j \in J}X_{ij}(T_{ij}-T_0)^+] \\&+ \sup _{G \in \overline{{\mathcal {G}}}} \mathbb {E}_G[\beta \sum _{i \in I}(D_i - \sum _{j \in J}X_{ij})^ +] \end{aligned}$$

*Subject to*

11 $$\begin{aligned}&N_j \le P \, Z_j \forall j \in J \end{aligned}$$

12 $$\begin{aligned}&\mu _{D_i} = \mathbb {E}_G(D_i) \le \sum _{j \in J} X_{ij} \forall i \in I \end{aligned}$$

13 $$\begin{aligned}&\sum _{i \in I} X_{ij} \le C \, N_j \forall j \in J \end{aligned}$$

14 $$\begin{aligned}&Z_j \in \{ 0, 1\}, \, N_j \in \mathbb {Z}^+ \forall j \in J \end{aligned}$$

15 $$\begin{aligned}&X_{ij} \in \mathbb {Z}^+ \forall i \in I, \, \forall j \in J. \end{aligned}$$

The purpose of the objective function (Eq. 10) is to minimize the supremum of the expected total cost by restricting the distributions of the random vector ***T*** (resp., ***D***) to the specified distributional set ${\mathcal {F}}$ (resp., ${\mathcal {G}}$). The total cost is the sum of the fire station construction costs, the purchase costs for fire trucks, the transportation cost, and the penalty cost for overtime and unsatisfied demands. Constraint (11) requires that fire trucks can only be assigned to open fire stations, and that the number of trucks placed in these stations does not exceed their capacity. Constraint (12) imposes that the average demand of every customer *i* is satisfied. Constraint (13) requires that the monthly total calls assigned by station *j* are no more than the number of assignments fire trucks placed at site *j* can handle. Constraint (14) imposes binary and non-negative integral restrictions on the decision variables.

### Reformulation of the worst-case expectation problem

The proposed DRM incorporates an optimization problem over distributions for demands and travel times within the ambiguity set. We reformulate these problems by dualizing the terms $\sup _{F \in \overline{{\mathcal {F}}}}\mathbb {E}_F[\theta \sum _{i \in I}\sum _{j \in J} T_{ij}X_{ij} + \eta \sum _{i \in I}\sum _{j \in J}X_{ij}(T_{ij}-T_0)^+] + \sup _{G \in \overline{{\mathcal {G}}}}\mathbb {E}_G[\beta \sum _{i \in I}(D_i - \sum _{j \in J}X_{ij})^ +]$ of objective (Eq. 10) so that they can be made into minimization problems. The methodological details are described in the first section of “Supplementary Appendix”. In addition, by further considering the primal decision variables ***Z***, ***N***, ***X***, we can recast Eq. (10) as the following finite-dimensional minimization problem:

16 $$\begin{aligned}&w_r^* = \min _ {\phi _1,\phi _2,{\varvec{{p}}},{\varvec{{q}}},{\varvec{{r}}},{\varvec{{s}}}} \sum _{j \in J} (Ms\cdot Z_j + Mv \cdot N_j) + \phi _1 + \phi _2 + {\varvec{{p}}}^T \varvec{\mu _T} + {\varvec{{q}}}^T \varvec{\sigma _T} + {\varvec{{r}}}^T \varvec{\mu _D} + {\varvec{{s}}}^T \varvec{\sigma _D} \end{aligned}$$

17 $$\begin{aligned}&s.t. {\varvec{{q}}} \ge 0, {\varvec{{s}}} \ge 0 \end{aligned}$$

18 $$\begin{aligned}&(\theta {\varvec{{X}}}^T - {\varvec{{p}}}_T) {\varvec{{T}}} + \eta {\varvec{{X}}}^T ({\varvec{{T}}} - T_0)^+ - {\varvec{{q}}}^T {\varvec{{v}}} \le \phi _1 \forall ({\varvec{{T}}},{\varvec{{v}}}) \in \overline{\mathbb {E}}_1 \end{aligned}$$

19 $$\begin{aligned}&\beta \sum _{i \in I} (D_i - \sum _{j \in J}X_{ij})^+ - {\varvec{{r}}}^T {\varvec{{D}}} - {\varvec{{s}}}^T {\varvec{{u}}} \le \phi _2 \forall ({\varvec{{D}}}, {\varvec{{u}}}) \in \overline{\mathbb {E}}_2 \end{aligned}$$

20 $$\begin{aligned}&(11)-(15) \end{aligned}$$

Constraints (18) and (19) can be regarded as robust constraints on the polytopic uncertainty sets $\overline{\mathbb {E}}_1$ and $\overline{\mathbb {E}}_2$. Additionally, the term $\eta {\varvec{{X}}}^T ({\varvec{{T}}} - T_0)^+$ in (18) is a convex piecewise linear function of ***T***, with a number of pieces equal to $2^m$, where m is equal to $|I|*|J|$. Likewise, the term $\sum _{i \in I} (D_i - \sum _{j \in J}X_{ij})^+$ in Eq. (19) is also a convex piecewise linear function over ***D*** with a number pieces equal to $2^n$, where n equals |*I*|. There are at least two different ways we could consider tackling constraints (18) and (19). In the first approach, because the constraints are piecewise linear and convex, we could turn them into an exponential number of linear inequalities. Creating robust equivalents to these constraints requires maximizing their left-hand-sides over the support sets $\overline{\mathbb {E}}_1$ and $\overline{\mathbb {E}}_2$, which can be achieved through the use of linear programming duality. This approach, however, necessitates the addition of an exponential number of constraints and variables. In the second approach, we could seek to optimize the left-hand-sides of constraints (18) and (19) directly over the support sets $\overline{\mathbb {E}}_1$ and $\overline{\mathbb {E}}_2$. This however corresponds to the solution of a convex maximization problem, for which closed form expressions are not easily obtained. The structure of the problem nevertheless suggests that it is sufficient to impose the constraints (18) and (19) for the extreme points of $\overline{\mathbb {E}}_1$ and $\overline{\mathbb {E}}_2$, as convex functions over polytopes are maximized at the extreme points of the polytope. This in turn suggests that a cutting plane algorithm might be suitable for the solution of this problem.

### Cutting-plane method

Since reformulated model Eqs. (16)–(20) can be regarded as a traditional robust mixed-integer linear optimization problem, it can be solved using the cutting plane method^38^. The specific procedure we implement is as follows:

1. Initialize Eq. (16) to be the nominal problem. It is defined similarly to (16) with the exception that sets of $\overline{\mathbb {E}}_1$ and $\overline{\mathbb {E}}_2$ are replaced with subsets of their elements, namely those that have values ${\varvec{{T}}} = \varvec{\mu }_{\varvec{{T}}}$, ${\varvec{{v}}}=0$, ${\varvec{{D}}}= \varvec{\mu }_{\varvec{{D}}}$ and ${\varvec{{u}}} = 0$.
2. MIO solver GUROBI 9.0 begins the branch-and-bound process to solve the nominal problem. Whenever an integer solution (${\varvec{{Z}}}_0$, ${\varvec{{N}}}_0$, ${\varvec{{X}}}_0$, $\phi _{1 _{0 }}$, $\phi _{2 _{0 }}$, ${\varvec{{p}}}_0$, ${\varvec{{q}}}_0$, ${\varvec{{r}}}_0$, ${\varvec{{s}}}_0$) is found, it is entered into the uncertain constraints (18) and (19) to determine whether it is cut off by elements of either $\overline{\mathbb {E}}_1$ and $\overline{\mathbb {E}}_2$. This can be determined by formulating the problem of maximizing the left-hand-side of Eq. (18) over the elements of $\overline{\mathbb {E}}_1$ and maximizing the left-hand-side of (19) over the elements of $\overline{\mathbb {E}}_2$. Both of these problems reduce to maximizing piecewise linear convex functions. They can therefore be formulated as integer programming subproblems and solved with GUROBI.
  - If any of the integer programming subproblems discovers a violated constraint, it is passed to the MIO solver as a new lazy constraint, which will remove the candidate integer solution from consideration. In particular, robust constraint (18) can be tackled through the following steps: Uncertain constraint (19) can be processed similarly. This ensures that only cuts active at integer solutions are added.
  - If no constraint is violated, the MIO solver accepts the integer solution as an incumbent “robust feasible” solution. The integrality gap is then evaluated relative to this solution. GUROBI continues the branch-and-bound process until either the integrality gap is sufficiently small or until the time limit is reached.

It is clear however that this approach requires the potential introduction of large number of constraints, which will likely translates into long computational times. As a result, it will likely be unsuitable for the solution of practical problems where there is a large number of demand sites. To circumvent this issue, we propose two additional methods to deal with the problem: an approximation reformulation by linear decision rules (LDRs), and a heuristic method that optimizes over a specific discrete probability distribution.

### Approximation by linear decision rules

Linear decision rules (LDRs) have been used in the optimization literature to create tractable approximations of robust optimization problems^39^. They have received less attention in the DRO literature^32,40,41^. In this section, we describe how they can be applied to the setting of our unique ambiguity set. This application has the attractive feature of keeping the model linear. Therefore, the reformulation will still take the form of a mixed-integer linear program.

Consider first the term $\sup _{F \in \overline{{\mathcal {F}}}}\mathbb {E}_F[\theta \sum _{i \in I}\sum _{j \in J} T_{ij}X_{ij} + \eta \sum _{i \in I}\sum _{j \in J}X_{ij}(T_{ij}-T_0)^+] + \sup _{G \in \overline{{\mathcal {G}}}}\mathbb {E}_G[\beta \sum _{i \in I}(D_i - \sum _{j \in J}X_{ij})^ +]$. This optimization model can be rewritten as:

$$\begin{aligned} \sup _{F \in \overline{{\mathcal {F}}}}\mathbb {E}_F[\theta \sum _{i \in I}\sum _{j \in J} T_{ij}X_{ij} + \eta \sum _{i \in I}\sum _{j \in J}q_{ij}(\mathbf{T} , \mathbf{v} )] + \sup _{G \in \overline{{\mathcal {G}}}}\mathbb {E}_G[\beta \sum _{i \in I}o_i(\mathbf{D} ,\mathbf{u} )] \\ \begin{aligned} s.t. &q_{ij}(T,v) \ge X_{ij}(T_{ij}-T_0)^+ \forall i \in I, j \in J, \forall ({\varvec{{T}}}, {\varvec{{v}}}) \in \overline{\mathbb {E}}_1 \\&o_i(\mathbf{D} ,\mathbf{u} ) \ge (D_i - \sum _{j \in J}X_{ij})^ + \forall i \in I, \forall (\mathbf{D} , \mathbf{u} ) \in \overline{\mathbb {E}}_2. \end{aligned} \end{aligned}$$

The overtime penalty cost $q_{ij}(\mathbf{T} , \mathbf{v} )$ is a function of $\mathbf{T}$ and $\mathbf{v}$, and the unsatisfied demand penalty cost $o_{i}(\mathbf{D} , \mathbf{u} )$ is a function $\mathbf{D}$ and $\mathbf{u}$. Accordingly, we can equivalently formulate the DRO model as follows:

21 $$\begin{aligned}&w_a^* = \min \sum _{j \in J} (Ms\cdot Z_j + Mv \cdot N_j) + \sup _{F \in \overline{{\mathcal {F}}}}\mathbb {E}_F[\theta \sum _{i \in I}\sum _{j \in J} T_{ij}X_{ij} + \eta \sum _{i \in I}\sum _{j \in J}q_{ij}(\mathbf{T} , \mathbf{v} )] + \sup _{G \in \overline{{\mathcal {G}}}}\mathbb {E}_G[\beta \sum _{i \in I}o_i(\mathbf{D} ,\mathbf{u} )] \end{aligned}$$

22 $$\begin{aligned}&s.t. q_{ij}(T,v) \ge X_{ij}(T_{ij}-T_0) \forall i \in I, j \in J, \forall ({\varvec{{T}}}, {\varvec{{v}}}) \in \overline{\mathbb {E}}_1 \end{aligned}$$

23 $$\begin{aligned}&q_{ij}(T,v) \ge 0 \forall i \in I, j \in J, \forall ({\varvec{{T}}}, {\varvec{{v}}}) \in \overline{\mathbb {E}}_1 \end{aligned}$$

24 $$\begin{aligned}&o_i(\mathbf{D} ,\mathbf{u} ) \ge D_i - \sum _{j \in J}X_{ij} \forall i \in I, \forall (\mathbf{D} , \mathbf{u} ) \in \overline{\mathbb {E}}_2 \end{aligned}$$

25 $$\begin{aligned}&o_i(\mathbf{D} ,\mathbf{u} ) \ge 0 \forall i \in I, \forall (\mathbf{D} , \mathbf{u} ) \in \overline{\mathbb {E}}_2 \end{aligned}$$

26 $$\begin{aligned}&(11)-(15). \end{aligned}$$

For tractability, we next adopt an LDR approach to tackle the penalty functions. For example, we restrict the overtime penalty cost for each demand site $i \in I$ and for each location $j \in J$ to be affine in ***T*** and ***v***, i.e.,

27 $$\begin{aligned} q_{ij}(\mathbf{T} ,\mathbf{v} ) = q_{ij}^0 + q_{ij}^1\mathbf{T} + q_{ij}^2\mathbf{v} \end{aligned}$$

where $q_{ij}^0$, $\mathbf{q} _{ij}^1$ and $\mathbf{q} _{ij}^2$ are decision variables, and we restrict the penalty for not satisfying the demand for each site $i \in I$ to be affine in ***D*** and ***u***, i.e.,

28 $$\begin{aligned} o_i(\mathbf{D} ,\mathbf{u} ) = o_i^0+o_i^1\mathbf{D} +o_i^2\mathbf{u} \end{aligned}$$

where $o_i^0$, $\mathbf{o} _i^1$ and $\mathbf{o} _i^2$ are also decision variables. Now the traditional robust counterpart technique can be used to convert model (Eq. 21) into a deterministic formulation. We do not present this transformation here but refer interested readers to the literature, e.g.,^32^. Instead, we use the Matlab RSOME tool^33^ to directly solve this model. The following proposition is easily proven based on the above statements.

#### Proposition 1

*Assuming these models have optimal solutions, the optimal value of the model Eqs.* (21)–(26) *reformulated by the approximation method yields an upper bound on the model Eqs.* (10)–(15), *i.e.*,

29 $$\begin{aligned} w^* \le w_a^*. \end{aligned}$$

It is easy to see that the objective value in Eq. (21) is larger than that in Eq. (10) because of the auxiliary variables $q_{ij}(\mathbf{T} ,\mathbf{v} )$ and $o_i(\mathbf{D} ,\mathbf{u} )$ are greater than the values they replace. Furthermore, the feasible region for Eqs. (21)–(26) is also no larger than that in Eqs. (10)–(15) because the approximation method only considers applying linear decision rules for $q_{ij}(\mathbf{T} ,\mathbf{v} )$ and $o_i(\mathbf{D} ,\mathbf{u} )$.

### Heuristic method via constructing a discrete distribution

In the DRM, the joint probability distribution *G* for random fire service demands and *F* for random travel durations are chosen from the ambiguity sets ${\mathcal {G}}$ and ${\mathcal {F}}$, respectively, in an adversarial fashion. This proves to be the primary source of difficulty in solving this model. To circumvent this difficulty, one could consider a heuristic method where a single discrete probability distribution is introduced in the model that approximates the worst-case distribution. Then, the problem complexity diminishes sharply as it yields a deterministic formulation without uncertainties. This heuristic method is based on the following proposition, which helps choose a marginal distribution for each random variable.

#### Proposition 2

^42^. *Given any convex function* *f(d)* *of a random variable* *d*, *and the ambiguity set* ${\mathcal {H}} = \{ P|\mathbb {E}_P(d) = \mu , \mathbb {E}_P(|d-\mu | = \sigma , P(d \in [\underline{d},\overline{d}]))=1 \}$ *of the distribution P. We have that*

30 $$\begin{aligned} \sup _{P \in {\mathcal {H}}}\mathbb {E}_Pf(d) = p_1f(\underline{d}) + p_2f(\mu ) + p_3f(\overline{d}), \end{aligned}$$

*where* $$p_1 = \frac{\sigma }{2(\mu -\underline{d})}$$, $$p_2 = 1 - \frac{\sigma }{2(\mu -\underline{d})}$$, *and* $$p_3 = \frac{\sigma }{2(\overline{d}-\mu )}$$.

This proposition suggests that the worst-case distribution in this situation is a three-points distribution on the lower bound $\underline{d}$, the mean $\mu$, and the upper bound $\overline{d}$ of the random variable *d*, with probability masses being $p_1$, $p_2$, and $p_3$, respectively. Furthermore, the values of probability masses can be computed from historical data.

Proposition 2 handles the case of a convex function of a single random variable *d*. We therefore believe it will work well for our case as the left-hand sides of constraints (18) and (19) are convex piecewise linear functions of random vector ***T*** and ***D***, respectively. The main idea of the heuristic method is to let the marginal distribution of each random variable $D_i(T_{ij})$ also be the three-point distribution. For more details, we refer the readers to the literature^32^. Finally, we can reformulate the proposed model into the form:

31 $$\begin{aligned}w_h^* = \min \sum _{j \in J} (Ms\cdot Z_j + Mv \cdot N_j)+ \mathbb {E}_{F_0}\left[ \theta \sum _{i \in I}\sum _{j \in J} T_{ij}X_{ij} + \eta \sum _{i \in I}\sum _{j \in J}X_{ij}(T_{ij}-T_0)^+\right] + \mathbb {E}_{G_0}\left[ \beta \sum _{i \in I}(D_i - \sum _{j \in J}X_{ij})^ +\right] \end{aligned}$$

32 $$\begin{aligned}&s.t. (11)-(15). \end{aligned}$$

Here, $F_0$ and $G_0$ represent the discrete distributions that are used to approximate the worst-case distributions of random variables ***T*** and ***D***, respectively. These distributions have a number of points in their supports that is polynomial with respect to the number of random variables; see^32^.

#### Proposition 3

*Assuming that these models have optimal solutions, the optimal value of Eqs.* (31)–(32) *constructed by the heuristic method yields a lower bound on the optimal value of Eqs.* (10)–(15), *i.e.*,

33 $$\begin{aligned} w_h^* \le w^* \end{aligned}$$

#### Proof

The objective function Eq. (10) is of the form $w^*=\min _{x,y}[f(x)+\max _{p \in Q}g(y,p)]$, where *x* and *y* represent decision variables, and *p* is a distribution belonging to the ambiguity set *Q*. Then, *f* is a linear function over *x*, and *g* is a piecewise linear function over *y* and *p*. Using this notation, the heuristic method formulation is $w_h^*=\min _{x,y}[f(x)+g(y,p_0)]$. Note that $p_0$ represents a discrete distribution that approximates the worst-case distribution *p* over *Q*. Suppose an optimal solution to the distributionally robust model is $(x^*,y^*,p^*)$ and an optimal solution to the heuristic formulation is $(x_h^*,y_h^*,p_0)$. Then,

34 $$\begin{aligned} w^* = f(x^*) + \max _{p \in Q} g(y^*,p) \ge f(x^*) + g(y^*,p_0) \ge f(x_h^*)+g(y_h^*,p_0) = w_h^* \end{aligned}$$

The first inequality holds because $p_0$ is one of the distributions in *Q* occuring in the maximum, and the second inequality holds because $(x^*,y^*)$ is one of the feasible solutions to the heuristic problem. We conclude that $w_h^* \le w^*$. This completes the proof.

## Data preparation

### Determination for the random weight of demand points

This paper chooses the city of Hefei (China) as the study area to examine the proposed model’s performance. The detailed records of fire accidents from 2002 to 2011 are used. These records are used because they are the most recent ones that are available to our research team. Further, the information they provide allow us to evaluate the quality and practicality of the proposed approach for instances encountered in practice. It is clear however that practitioners might obtain more up-to-date prescriptions using data from later years.

**Figure 2:**
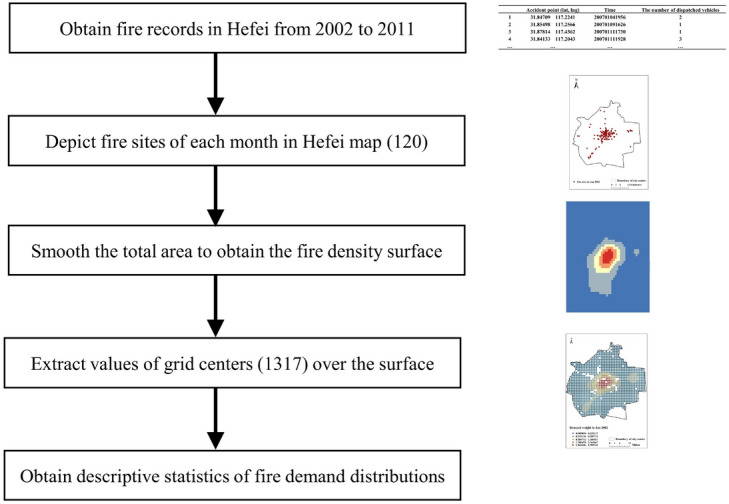
Process for acquiring fire demand distributions.

**Figure 3:**
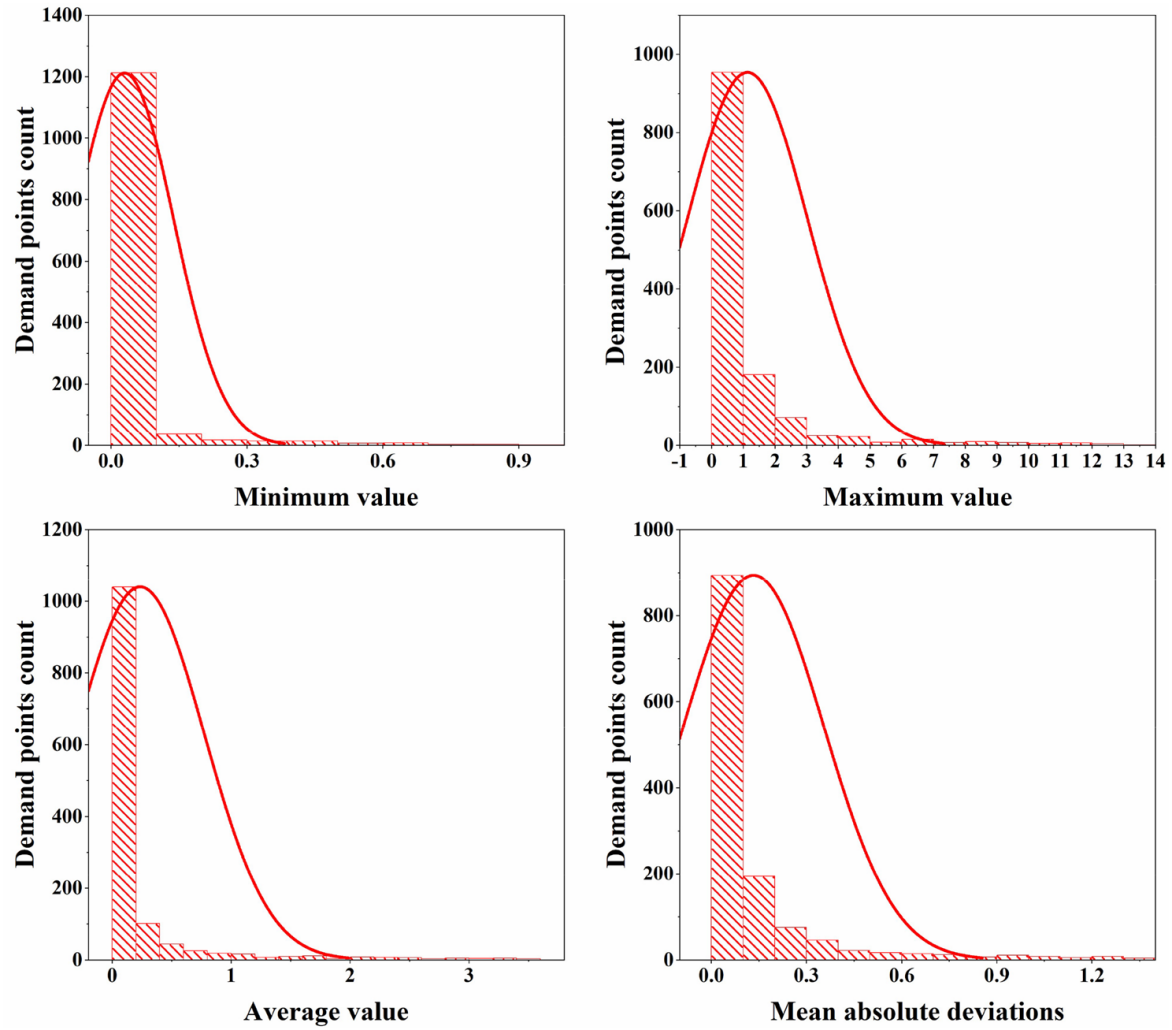
Descriptive statistics of demand weights from 2002 to 2011.

The records we use contain information about all fire accidents that occur within the study period including site, request time, and number of dispatched fire trucks, among others. After processing this historical data, 120 monthly average demand weights can be obtained for the entire area. Specifically, the fire accidents for each month are first depicted on the map of Hefei. The kernel density estimation tool in GIS is then used to smooth the total area based on the demand weight (the number of fire trucks dispatched) of those fire incidents. Finally, after conducting the “Extract value to points” operation in GIS, a total of 1317 grid center points are generated with their demand weight (monthly average fire trucks required) each month. Because of the way they are constructed, demand weights need not be integer. The corresponding process is shown in Fig. 2. In addition, Fig. 3 shows the descriptive statistics of the demand weights from 2002 to 2011.

### Determination of the random travel durations

**Figure 4:**
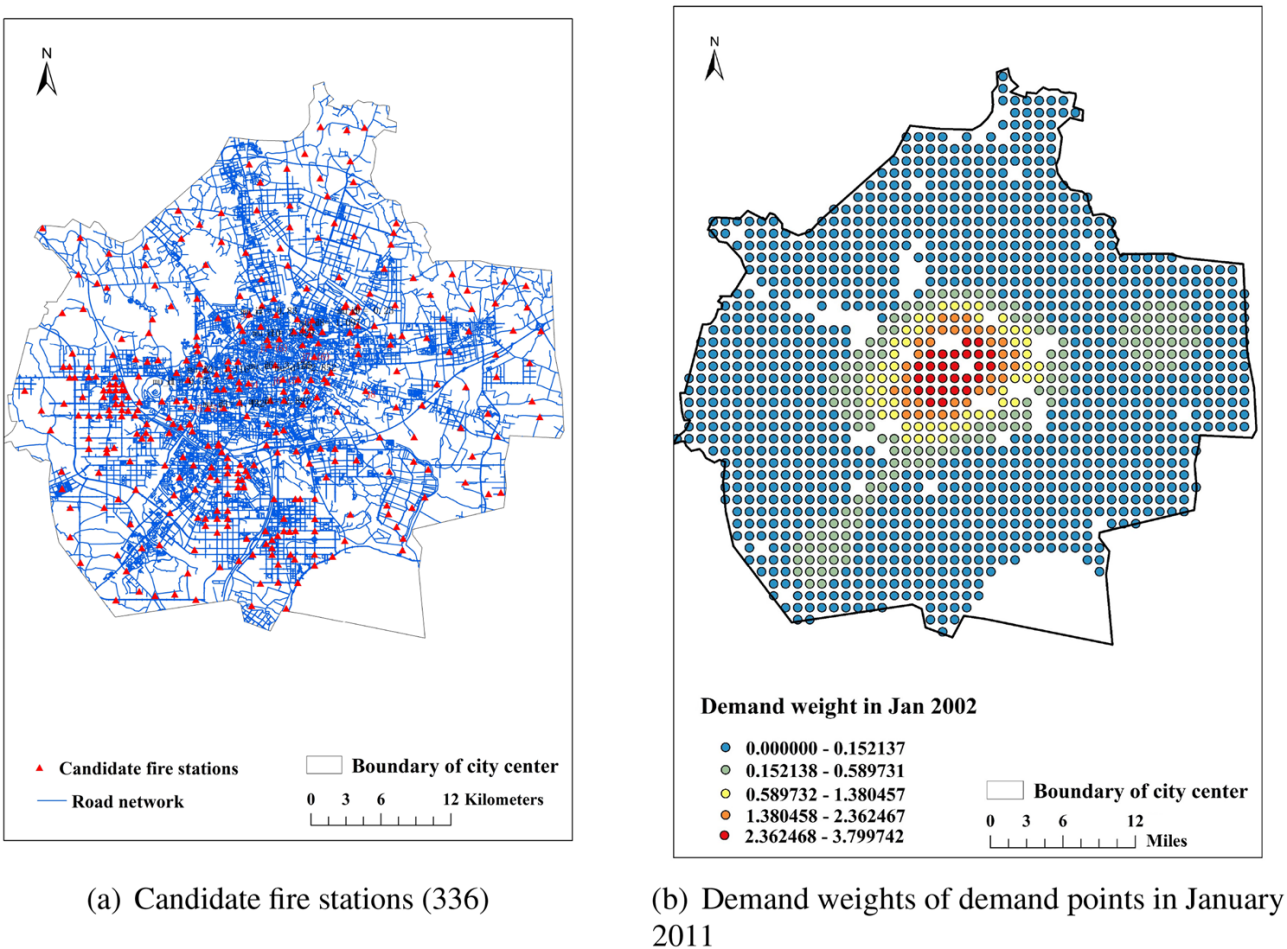
Distribution of potential fire stations and demand points.

Before estimating descriptive statistics of the distributions of random travel times, the locations of candidate fire stations must first be selected. This is achieved by considering the primary road network and by using the GIS function and the network analysis tool. The initial candidate sites for fire stations are selected to be all of the nodes in the road network dataset. Then, the analysis function of “buffer calculation” and “attribute selection” makes it possible to remove the sites that are relatively close to existing fire stations and POIs (points of interests), where congestion is assumed to be large and therefore to hamper rapid response times. This procedure produces 336 potential fire stations locations. Finally, the Gaode API is used to crawl the traffic time in six periods (including weekday morning peak period, weekday evening peak period, weekday off-peak period, weekend morning peak period, weekend evening peak period, and weekend off-peak period) from all of the candidate fire stations (336) to all of demand points (1317). Using the six travel time tables obtained in this way, descriptive statistics, including minimum value, maximum value, average value, and mean absolute deviation are obtained for each random variable $T_{ij}$. Figure 4 presents candidate fire stations together with the distribution of demand points (shown as the demand weights) for January 2011.

## Computational experiments

In this section, a first computational experiment is performed to compare the performance of the three proposed approaches and to analyze the influence of the parameters used. Then a real-sized scenario is considered with the goal of validating the practical applicability and value of the proposed DRO location model. Our experiments are carried out on a Lenovo Y7000 PC, with an Intel (R) Core (TM) i7-9750H CPU running at 2.60 GigaHertz and 16.00 Gigabyte of memory. All of the instances are solved with the optimization solver GUROBI. It is set to terminate either when a relative MIP optimality gap smaller than 1% has been achieved, or when a maximum running time set to one hour has been exceeded. These results are analyzed in the following subsections.

### Basic experiments

The parameters of the following experiments are chosen from the values listed in Table 3. The values of some of these parameters are fixed. For example, the purchase price of one fire truck is assumed to be ¥250,000, following fire department records. The construction cost of one fire station is set as ¥5,000,000 based on the records from both the fire department and related references^15,43^. The unit transportation $\theta$ cost is ¥50,000, which includes the property loss caused by the fire accident. The influence of the other parameters, such as $\eta$, $\beta$, $T_0$, and *P*, on the solution of the DRO model is investigated by considering multiple values for them.

**Table 3:** Test values for the parameters expressed in Eq. (¥10,000) unless otherwise specified.

Parameters	Value
$\theta$	5
$\eta$	[10, 20, 30, 50, 80, 100]
$\beta$	[10, 50, 80, 100, 150, 200]
$T_0$	[2, 4, 6, 10, 12 15] min
*P*	6
*Ms*	500
*Mv*	25

The first computational experiment seeks to evaluate how the performance of the three methods changes as a function of the number of demand points and candidate locations. For various selected sizes, ten instances are created by selecting demand points and candidate locations randomly from the practical data set. Computational times are listed in Table 4. The number in the brackets reports the average computing time across all generated instances. The unit is seconds. It can be observed that the heuristic method performs best with regard to computational time for every instance. Second is the approximation method by LDRs. The cutting plane method has the worst performance.

Additionally, all of the methods report the same objective value for each instance of small size. This suggests that, for these instances generated from real recorded data, the heuristic and the approximation methods, even though they are heuristic, might often generate solutions of the same quality as the exact method. The results also show that the heuristic method is likely the only method we investigated that has the potential for solving larger-scale instances of the problem. As explained in the Model formulation section, the heuristic method provides a lower bound and the approximation method using LDRs provides an upper bound to the optimal value of the problem. It follows that, for all instances solved within one hour, both of these methods generate an optimal solution to the problem; for those instances solved up to an hour, acceptable objective bounds are found in the cutting plane method and the approximation method. When the cutting plane approach terminates because the time limit has been reached, the incumbent solution it returns is a solution that meets constraints (11)–(15) but may not satisfy some of the constraints (18)–(19). It therefore yields a lower bound on the optimal value of the problem. Intuitively, it is a solution that is robust with respect to many scenarios in the uncertainty set but not to all of them. When the LDR approach terminates because the time limit has been reached, it provides an overestimation of the cost incurred in the system that is not the best possible such estimation. It therefore provides an upper bound on the optimal value of the problem.

**Table 4:** Performance of the three methods (($\theta , \eta , \beta , P, T_0) =$ (5, 50, 100, 6, 6)).

Instance (|*I*|, |*J*|)	Heuristic method (lower bound)	Cutting plane (exact solution)	Approximation by LDRs (upper bound)
(10, 8)	1835.14 (0.2 s)	1835.14 (22.57 s)	1835.14 (3.5 s)
(20, 15)	2749.75 (2.16 s)	2596.73 (1 h)	2749.75 (65.36 s)
(30, 20)	3420.79 (8.61 s)	3091.09 (1 h)	3420.79 (412.2 s)
(100. 80)	7701.38 (118.21 s)	6947.53 (1 h)	7935.22 (1 h)

The second experiment seeks to evaluate the influence of the cost parameters $\eta$ and $\beta$ on the optimal value and optimal solutions of the problem. It can be observed in Table 5 that higher values of $\eta$ cause a slower increasing rate for the total cost. Besides, when the per-unit penalty cost $\eta$ for overtime is less than 80, the pure cost (construction fee of building fire stations plus purchase cost of fire trucks) is constant. This cost then increases by the purchase cost of a single fire engine and the construction cost of one fire station when $\eta$ is over 80. Moreover, the total cost almost linearly increases with the per-unit penalty cost $\beta$ for unsatisfied demands. The pure cost also keeps constant when $\beta$ is no more than 150. Accordingly, it can be concluded that small changes in the unit penalty cost $\eta$ and $\beta$ have little impact on the pure cost representing the optimal fire station sites and the assignment of fire trucks. A possible explanation for this behavior is that the proposed model considers the worst-case of random demands and random travel durations and therefore settles on similar configurations.

**Table 5:** Influence of the cost parameters (($|I|, |J|, P, T_0$) = (10, 8, 6, 6)).

$(\theta , \eta , \beta )$	Objective value	$(\theta , \eta , \beta )$	Objective value
Instance1 (5, 10, 100)	1413.65 (525, ‘j1’)	Instance7 (5, 50, 10)	1542.05 (525,’j1’)
Instance2 (5, 20, 100)	1519.02 (525,’j1’)	Instance8 (5, 50, 50)	1673.76 (525,’j1’)
Instance3 (5, 30, 100)	1624.4 (525,’j1’)	Instance9(5, 50, 80)	1772.55 (525,’j1’)
Instance4 (5, 50, 100)	1835.14 (525,’j1’)	Instance10 (5, 50, 100)	1835.14 (525,’j1’)
Instance5 (5, 80, 100)	1956.06 (1050’j1’,’j4’)	Instance11 (5, 50, 150)	1971.35 (525,’j1’)
Instance6 (5, 100, 100)	2013.66 (1050’j1’,’j4’)	Instance12 (5, 50, 200)	2061.37 (1050’j1’,’j4’)

The number in the bracket in the second column and the fourth column represents the pure cost. The letters in the bracket shows which site to open a fire station.

The third experiment seeks to evaluate the influence of the standard fire response time $T_0$. Table 6 shows that lower cost can be realized by setting higher response time for every single opened fire station. This is intuitively clear because increasing the standard response time can reduce the penalty cost for overtime. When the response time $T_0$ is large enough, however, the pure cost becomes stable as the model is able to select an optimal number of trucks for each station. For example, in cases 1 and 2, this happens when the value of $T_0$ is at least 6; in case 3, this happens when the value of $T_0$ is at least 10. Therefore, based on a limited budget, even though the fire response time is uncertain and sometimes higher than usual, the layout of the optimal sites and fire trucks has a reliable fire rescue performance.

**Table 6:** Influence of the standard fire response time $T_0$ when ($\theta , \eta , \beta$) = (5, 50, 100)).

$T_0(P=6)$	Case1	Case2	Case3
	$(|I|=10,|J|=8)$	$(|I|=20,|J|=15)$	$(|I|=30,|J|=20)$
2	3181.66 (3, 3, 1575)	5228.96 (5, 5, 2625)	6064.45 (4, 5, 2125)
4	2249.09 (2, 2, 1050)	3641.1 (3, 3, 1575)	4239.3 (4, 5, 2125)
6	1835.14 (1, 1, 525)	2749.75 (2, 2, 1050)	3420.79 (3, 4, 1600)
10	1379.04 (1, 1, 525)	2199.74 (2, 2, 1050)	2788.72 (2, 3, 1075)
12	1337.32 (1, 1, 525)	2090.21 (2, 2, 1050)	2749.52 (2, 3, 1075)

The data in the last three columns is presented as Total cost (Station count, Vehicle count, Pure cost).

Because solving large-scale instances of our model can be challenging, we next provide a strategy to decrease its size sharply. Two kinds of random variables are present in the model: random demand and uncertain travel time. In the practical data set, there exist 1317 demand points and 336 candidate fire stations. If it is necessary to consider every pair of them, there should be 1317*336 random travel time variables. This size is too large to deal with it. A simple idea to circumvent the challenge is to consider a certain number of nearest candidate fire stations for every demand point. We call this number the *neighbor count* (Nc). Table 7 presents relationships between the Nc and the objective value for four kinds of instances. They are tested on a cloud server with a 128.00 GB of memory and solved with a time limit of 2 h. It is easy to find that when the size of the instance grows, Nc should also increase to obtain a superior solution or even an optimal solution. For example, the optimal value for the three instances stabilizes when Nc is equal to 10, 20, and 20, respectively. For the last real-sized case, Nc should likely be more than 20 for an optimal solution to be obtain. However, given the computing time cost and server’s memory limitation, this paper selects Nc to be 15 in the next section, from which we expect to obtain high quality feasible solutions.

**Table 7:** Influence of the neighbor count for each demand point (($\theta , \eta , \beta , P, T_0$) = (5, 50, 100, 6, 6)).

Neighbor count (Nc)	Objective value	Neighbor count (Nc)	Objective value
$(|I|=30,|J|= 20)$		$(|I|=100,|J|=80)$	
5	3532.34	5	10739.5
10	3420.79	10	8354.35
15	3420.79	15	7964.1
20	3420.79	20	7701.38
25	3420.79	25	7701.38
Neighbor count (Nc)	Objective value	Neighbor count (Nc)	Objective value
$(|I|=658,|J|= 336)$		$(|I|=1317,|J|=336)$	
5	49738.2	5	66817.3
10	38430.7	10	55762.34
15	35477.9	15	52848.58
20	34232.4	20	Out of memory
25	34232.4	25	Out of memory

### Extended experiments

This subsection includes two experiments. In the first, we seek to compare the performance of the DRO model with that of a stochastic programming (SP) model shown in the Supplementary Appendix. In the second, we seek to evaluate the quality and practicality of the heuristic approach for solving real-life instances of the problem.

We propose to compare the performance of these approaches by using a subset of the available historical data to populate distributions and ambiguity sets in the models, and to use the rest of the historical data to compute performance measures for the prescriptions obtained from the different models. In particular, we partition the historical data into two subsets for in-sample training and out-of-sample evaluation. Data from 2002 to 2010 is used for training. Data from 2011 is used to evaluate the out-of-sample performance of the solutions. For each scenario/instance, the total cost (composed of construction costs, purchase costs for fire trucks, transportation cost, and two kinds of penalty costs) is calculated. The following indicators, which are of interest to local authorities, are reported:

- Aver: average out-of-sample cost including construction fee of building fire stations, purchase cost of fire trucks, and transportation cost.
- Qua: upper-quartile (75th percentile) out-of-sample cost (defined as in Aver.)
- Wor: worst out-of-sample cost (defined as in Aver.)
- Time: computational time required for solving the instance.

**Figure 5:**
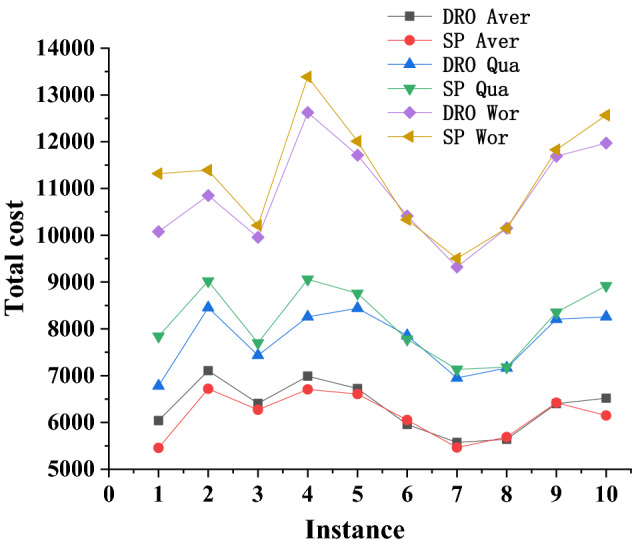
Comparison of the DRO and SP solutions.

The first experiment seeks to compare the solutions obtained from the DRO model with those obtained from the stochastic program. For computational tractability, we consider small-sized instances of the problem with $|I|=30$ demand points and $|J|=20$ candidate fire station locations. We also set Nc to be infinite. These instances are generated by selecting, from the real-life data set, random demand points and candidate locations from the center of Hefei. Ten instances are tested and associated performance indicators are computed. Table 8 and Fig. 5 report the computational results. The last row of Table 8 presents the average relative GAP between indicators (*Aver*, *Qua*, *Wor*) for the DRO model and for the SP model. We compute the relative GAP between indicators using the formula (indicator of DRO model − indicator of SP model)/(indicator of DRO model). Obviously, the more negative the relative GAP of an indicator, the better the DRO model performs. Hence, if the relative *GAP* is positive, the SP model outperforms the DRO model.

From the computational results, it can be observed that the SP approach outperforms the DRO model in terms of average costs (*Aver*), as it produces a solution that is about 3% better. This was expected since the DRO model only uses limited descriptive statistics of the ambiguity set, whereas the SP model considers the entire empirical distribution. However, in terms of worst-case performance *Wor* and upper-quartile performance *Qua*, the DRO model performs much better than SP. Figure 5 illustrates this point graphically. For the *Aver* indicator, the curve for the SP model is under that for the DRO model. For the other two indicators *Wor* and *Qua*, the curves for the DRO model are for the most part under the corresponding curves for the SP model. Therefore, the DRO solution, although more conservative, might be desirable for risk-averse decision makers who want to guard against extreme scenarios.

**Table 8:** Comparison of the DRO and SP solutions (($|I|,|J|,\theta , \eta , \beta , P, T_0) =$ (30, 20, 5, 50, 100, 6, 6)).

Instance	Distributionally robust solutions			Stochastic programming solutions		
	Aver	Qua	Wor	Aver	Qua	Wor
1	6038.82	6778.95	10078.87	5454.87	7841.17	11317.41
2	7106.67	8452.80	10849.75	6719.69	9020.0	11392.93
3	6406.40	7434.98	9955.88	6271.33	7700.59	10214.05
4	6989.95	8257.28	12623.93	6705.60	9060.09	13386.91
5	6723.16	8439.64	11712.76	6607.57	8759.07	12008.43
6	5956.75	7857.03	10413.08	6050.86	7777.00	10333.44
7	5574.20	6950.11	9322.88	5464.66	7133.70	9505.58
8	5637.95	7165.05	10152.06	5687.37	7183.34	10151.81
9	6400.54	8207.09	11689.96	6421.25	8353.94	11827.79
10	6518.17	8257.43	11970.43	6149.01	8922.48	12566.17
**GAP(%)**	2.87	−5.07	−3.62	–	–	–

**Table 9:** Performance of the model for the entire study area (($\theta , \eta , \beta , P, T_0) =$ (5, 50, 100, 6, 6)).

Scenarios	Performance			
	Aver	Qua	Wor	Time (s)
Base	49666.656	50239.598	50456.174	4425.63
Instance 1(1)	49302.510	49877.596	50136.518	4646.42
Instance 2 (2)	48728.090	49308.452	49522.041	4484.26
Instance 3 (4)	47756.093	48365.738	48506.027	4418.10

The number in the bracket in the first column represents the number of reconstructed fire stations.

The other experiment considers a large-scale instance arising in a practical scenario with 1317 demand points and 336 candidate fire stations. Based on the results mentioned above, it seems impractical to utilize the approximation method by LDRs and the cutting plane method to solve this case within an acceptable time. Accordingly, we adopt the heuristic method, impose a computation time limit of 2 h for solving the model, and set the value of Nc equal to 15. To evaluate the quality of our approach, a baseline situation is first computed. In this baseline, the model is optimized under the assumption that the fire stations built are the 32 fire stations present in Hefei in 2008. Then, three scenarios are studied where 1, 2, and 4 established fire stations are rebuilt, respectively.

Computational results are presented in Table 9. The last instance (rebuilding four fire stations) performs the best on all of the performance indicators (Aver, Qua, and Wor) except for the computing time. Clearly, costs could be further reduced by increasing the number of reconstructed stations. For example, for the worst-case performance (Wor), about ¥9.34 millions could be saved by rebuilding two fire stations, but just ¥3.19 millions for reconstructing one fire station. The problem of deciding whether the costs savings warrant the actual construction of new fire stations, which local authorities must answer, is not one that will be addressed in this paper. The last column represents the computational time. All the instances take about 1.2 h to solve, which is acceptable for this application. This implies that the presented DRO model has the potential to be used in practice by local government seeking to locate fire stations.

## Conclusion and future work

This research aims to support urban planners in developing long-term EFS system designs, including fire station locations and fire trucks pre-positioning decisions that enable efficient fire service responses under uncertain demands and uncertain travel durations. A DRO model for fire station location and sizing problems is proposed to handle the inherent uncertainties in EFS systems and to design a reliable EFS network. Three approaches for the solution of this model are introduced, including a cutting-plane method, a heuristic method, and an approximation method using LDRs. The computational characteristics and performance of these approaches are compared. Sensitivity to the parameters of the DRO model is also evaluated.

Based on extensive numerical experiments, the following conclusions can be drawn. First, in terms of the different solution approaches, the heuristic method achieves the best performance, especially in computing time. Second, the numerical experiments show that the DRO model can ensure system reliability when facing demand uncertainties and travel time uncertainties. Third, the practical dataset from Hefei Fire Bureau demonstrates the practical applicability and value of the proposed data-driven method.

For future research, highly effective algorithms could be developed to tackle large-scale instances of the DRO model. It would be interesting to adopt other ambiguity sets and compare the results they yield. Also, an integrated DRO model considering other characteristics, such as dispatching various types of vehicles for different kinds of customers, could be studied to obtain more detailed management insights for the local Fire Bureau. Another possible direction is to consider participants’ behavior in EFS systems, e.g., decision-makers’ risk attitudes.

## Supplementary Information

Supplementary Information.
