# Supplementary material for: Distributionally robust optimization for fire station location under uncertainties
Source: Sci Rep. 2022 Mar 30;12:5394. doi: 10.1038/s41598-022-08887-6 (PMC8967840; doi:10.1038/s41598-022-08887-6)
Supplement: Supplementary file 1 — Supplementary Information. [file 41598_2022_8887_MOESM1_ESM.pdf]

Appendix

Proof for the reformulation of the worst-case expectation problem

Based on the definition of objective function (10), an explicit expression for the worst-case expectation problem is given by:

$$\sup_{F\in\overline{\mathscr{F}}} \mathbb{E}_F[\theta\sum_{i\in I}\sum_{j\in J} T_{ij}X_{ij} + \eta\sum_{i\in I}\sum_{j\in J} X_{ij}(T_{ij}-T_0)^+] + \sup_{G\in\overline{\mathscr{G}}} \mathbb{E}_G[\beta\sum_{i\in I}(D_i - \sum_{j\in J} X_{ij})^+]$$

$$s.t.$$

$$\int_{\overline{\mathbb{E}}_1} dF(\mathbf{T},\mathbf{v}) = 1$$

$$\int_{\overline{\mathbb{E}}_2} dF(\mathbf{D},\mathbf{u}) = 1$$

$$\int_{\overline{\mathbb{E}}_1} T_{ij}\, dF(\mathbf{T},\mathbf{v}) = \mu_{T_{ij}} \quad \forall i\in I, \forall j\in J$$

$$\int_{\overline{\mathbb{E}}_1} v_{ij}\, dF(\mathbf{T},\mathbf{v}) = \sigma_{T_{ij}} \quad \forall i\in I, \forall j\in J$$

$$\int_{\overline{\mathbb{E}}_2} D_i\, dG(\mathbf{D},\mathbf{u}) = \mu_{D_i} \quad \forall i\in I$$

$$\int_{\overline{\mathbb{E}}_2} u_i\, dG(\mathbf{D},\mathbf{u}) = \sigma_{D_i} \quad \forall i\in I$$

$$dF(\mathbf{T},\mathbf{v}) \geq 0$$

$$dG(\mathbf{D},\mathbf{u}) \geq 0.$$

Here the decision variables are the joint probability density function $dF(\boldsymbol{T},\boldsymbol{v})$ and $dG(\boldsymbol{D},\boldsymbol{u})$ or the probability measure F and G. By associating Lagrange multipliers ϕ_1, ϕ_2, $\boldsymbol{p}$, $\boldsymbol{q}$, $\boldsymbol{r}$ and $\boldsymbol{s}$ with the constraints, we obtain the following Lagrangian

$$L(F,G,\phi_1,\phi_2,\boldsymbol{p},\boldsymbol{q},\boldsymbol{r},\boldsymbol{s}) = \int_{\overline{\mathbb{E}}_1} [\theta\boldsymbol{X}^T\boldsymbol{T} + \eta\boldsymbol{X}^T(\boldsymbol{T}-T_0)^+]dF(\boldsymbol{T},\boldsymbol{v}) + \phi_1(1-\int_{\overline{\mathbb{E}}_1} dF(\boldsymbol{T},\boldsymbol{v})) + \boldsymbol{p}^T(\boldsymbol{\mu}_T - \int_{\overline{\mathbb{E}}_1} \boldsymbol{T}dF(\boldsymbol{T},\boldsymbol{v}))$$

$$+\boldsymbol{q}^T(\boldsymbol{\sigma}_T - \int_{\overline{\mathbb{E}}_1} \boldsymbol{v}dF(\boldsymbol{T},\boldsymbol{v})) + \int_{\overline{\mathbb{E}}_2} [\beta\sum_{i\in I}(D_i - \sum_{j\in J} X_{ij})^+]dG(\boldsymbol{D},\boldsymbol{u})) + \phi_2(1-\int_{\overline{\mathbb{E}}_2} dG(\boldsymbol{D},\boldsymbol{u}))$$

$$+\boldsymbol{r}^T(\boldsymbol{\mu_D} - \int_{\overline{\mathbb{E}}_2} \boldsymbol{D}dG(\boldsymbol{D},\boldsymbol{u})) + \boldsymbol{s}^T(\boldsymbol{\sigma_D} - \int_{\overline{\mathbb{E}}_2} \boldsymbol{u}dG(\boldsymbol{D},\boldsymbol{u}))$$

$$= \phi_1 + \phi_2 + \boldsymbol{p}^T\boldsymbol{\mu_T} + \boldsymbol{q}^T\boldsymbol{\sigma_T} + \boldsymbol{r}^T\boldsymbol{\mu_D} + \boldsymbol{s}^T\boldsymbol{\sigma_D} + \int_{\overline{\mathbb{E}}_1} [(\theta\boldsymbol{X}^T - \boldsymbol{p}_T)\boldsymbol{T} + \eta\boldsymbol{X}^T(\boldsymbol{T}-T_0)^+ - \phi_1 - \boldsymbol{q}^T\boldsymbol{v}]dF(\boldsymbol{T},\boldsymbol{v})$$

$$\int_{\overline{\mathbb{E}}_2} [\beta\sum_{i\in I}(D_i - \sum_{j\in J} X_{ij})^+ - \phi_2 - \boldsymbol{r}^T\boldsymbol{D} - \boldsymbol{s}^T\boldsymbol{u}]dG(\boldsymbol{D},\boldsymbol{u})$$

The lagrangian dual can then be written as:

$$g(\phi_1,\phi_2,\boldsymbol{p},\boldsymbol{q},\boldsymbol{r},\boldsymbol{s}) = \sup_{F,G} L(F,G,\phi_1,\phi_2,\boldsymbol{p},\boldsymbol{q},\boldsymbol{r},\boldsymbol{s}) = \phi_1 + \phi_2 + \boldsymbol{p}^T\boldsymbol{\mu_T} + \boldsymbol{q}^T\boldsymbol{\sigma_T} + \boldsymbol{r}^T\boldsymbol{\mu_D} + \boldsymbol{s}^T\boldsymbol{\sigma_D},$$

if $(\theta\boldsymbol{X}^T - \boldsymbol{p}_T)\boldsymbol{T} + \eta\boldsymbol{X}^T(\boldsymbol{T}-T_0)^+ - \phi_1 - \boldsymbol{q}^T\boldsymbol{v} \leq 0, \forall(\boldsymbol{T},\boldsymbol{v}) \in \overline{\mathbb{E}}_1$, and $\beta\sum_{i\in I}(D_i - \sum_{j\in J} X_{ij})^+ - \phi_2 - \boldsymbol{r}^T\boldsymbol{D} - \boldsymbol{s}^T\boldsymbol{u} \leq 0, \forall(\boldsymbol{D},\boldsymbol{u}) \in \overline{\mathbb{E}}_2$. In fact, if there exists $(\boldsymbol{T}^*,\boldsymbol{v}^*) \in \overline{\mathbb{E}}_1$, $(\boldsymbol{D}^*,\boldsymbol{u}^*) \in \overline{\mathbb{E}}_2$ such that $(\theta\boldsymbol{X}^T - \boldsymbol{p}_T)\boldsymbol{T}^* + \eta\boldsymbol{X}^T(\boldsymbol{T}^*-T_0)^+ - \phi_1 - \boldsymbol{q}^T\boldsymbol{v}^* \geq 0$ and $\beta\sum_{i\in I}(D_i^* - \sum_{j\in J} X_{ij})^+ - \phi_2 - \boldsymbol{r}^T\boldsymbol{D}^* - \boldsymbol{s}^T\boldsymbol{u}^* \geq 0$, then the dual function $g(\phi_1,\phi_2,\boldsymbol{p},\boldsymbol{q},\boldsymbol{r},s)$ will be unbounded because $dF(\mathbf{T},\mathbf{v}) \geq 0$, $dG(\mathbf{D},\mathbf{u}) \geq 0$.

Using these observations, we conclude that the dual model of the worst-case expectation problem can be written as

$$\min_{\phi_1,\phi_2,\boldsymbol{p},\boldsymbol{q},\boldsymbol{r},\boldsymbol{s}} \phi_1 + \phi_2 + \boldsymbol{p}^T\boldsymbol{\mu_T} + \boldsymbol{q}^T\boldsymbol{\sigma_T} + \boldsymbol{r}^T\boldsymbol{\mu_D} + \boldsymbol{s}^T\boldsymbol{\sigma_D}$$

$$s.t. (\theta\boldsymbol{X}^T - \boldsymbol{p}_T)\boldsymbol{T} + \eta\boldsymbol{X}^T(\boldsymbol{T}-T_0)^+ - \boldsymbol{q}^T\boldsymbol{v} \leq \phi_1 \quad \forall(\boldsymbol{T},\boldsymbol{v}) \in \overline{\mathbb{E}}_1$$

$$\beta\sum_{i\in I}(D_i - \sum_{j\in J} X_{ij})^+ - \boldsymbol{r}^T\boldsymbol{D} - \boldsymbol{s}^T\boldsymbol{u} \leq \phi_2 \quad \forall(\boldsymbol{D},\boldsymbol{u}) \in \overline{\mathbb{E}}_2,$$

$$\boldsymbol{q} \geq 0, \boldsymbol{s} \geq 0.$$

For this conic optimization problem, strong duality holds[1], and hence the optimal value of the dual problem is identical to that of the primal problem.

Stochastic programming model

In the stochastic fire station location problem, demands and travel durations are modeled as random variables $\mathbf{T} = (T_{ij})_{i\in I, j\in J}$ and $\mathbf{D} = (D_i)_{i\in I}$ with given probability distributions F_s and G_s, respectively. The model is given as follows:

$$w_S^* = \min \sum_{j\in J}(Ms\cdot Z_j + Mv\cdot N_j) + \mathbb{E}_{F_S}[\theta\sum_{i\in I}\sum_{j\in J}T_{ij}X_{ij} + \eta\sum_{i\in I}\sum_{j\in J}X_{ij}(T_{ij}-T_0)^+] + \mathbb{E}_{G_S}\beta\sum_{i\in I}(D_i - \sum_{j\in J}X_{ij})^+$$

$$s.t. \qquad (11)-(15).$$

This stochastic programming model is often intractable[2]. Thus, Denton et al.[3] suggest using the sample average approximation technique to reformulate it as follows:

$$w_S^* = \min \sum_{j\in J}(Ms\cdot Z_j + Mv\cdot N_j) + K_1^{-1}\sum_{\omega_1\in\Omega_1}[\theta\sum_{i\in I}\sum_{j\in J}T_{ij}^{\omega_1}X_{ij} + \eta\sum_{i\in I}\sum_{j\in J}X_{ij}(T_{ij}^{\omega_1}-T_0)^+] + K_2^{-1}\sum_{\omega_2\in\Omega_2}\beta\sum_{i\in I}(D_i^{\omega_2} - \sum_{j\in J}X_{ij})^+$$

$$s.t. \qquad (11)-(15).$$

Here, ω_1 and ω_2 represent sample indices, while Ω_1 and Ω_2 denote the set of all indices. Specifically, $T_{ij}^{\omega_1}$ represents the travel duration from fire station $j \in J$ to demand site $i \in I$ in sample $\omega_1 \in \Omega_1 = \{1,2,\ldots,K_1\}$. Similarly, $D_i^{\omega_2}$ represents the demand for site $i \in I$ in sample $\omega_2 \in \Omega_2 = \{1,2,\ldots,K_1\}$. By introducing variables $q_{ij}^{\omega_1}$ and $o_i^{\omega_2}$ that capture the overtime for travel time T_{ij} in sample $\omega_1 \in \Omega_1$ and the shortage for demand service D_i in sample $\omega_2 \in \Omega_2$, the problem can be further reformulated as a mixed integer linear programming model:

$$w_S^* = \min \sum_{j\in J}(Ms\cdot Z_j + Mv\cdot N_j) + K_1^{-1}\sum_{\omega_1\in\Omega_1}[\theta\sum_{i\in I}\sum_{j\in J}T_{ij}^{\omega_1}X_{ij} + \eta\sum_{i\in I}\sum_{j\in J}q_{ij}^{\omega_1}] + K_2^{-1}\sum_{\omega_2\in\Omega_2}\beta\sum_{i\in I}o_i^{\omega_2}$$

$$s.t. \qquad q_{ij}^{\omega_1} \geq X_{ij}(T_{ij}^{\omega_1} - T_0) \quad \forall i\in I, j\in J, \omega_1\in\Omega_1, \forall(\boldsymbol{T},\boldsymbol{v})\in\overline{\mathbb{E}}_1$$

$$q_{ij}^{\omega_1} \geq 0 \quad \forall i\in I, j\in J, \omega_1\in\Omega_1, \forall(\boldsymbol{T},\boldsymbol{v})\in\overline{\mathbb{E}}_1$$

$$o_i^{\omega_2} \geq D_i^{\omega_2} - \sum_{j\in J}X_{ij} \quad \forall i\in I, \omega_2\in\Omega_2, \forall(\mathbf{D},\mathbf{u})\in\overline{\mathbb{E}}_2$$

$$o_i^{\omega_2} \geq 0 \quad \forall i\in I, \omega_2\in\Omega_2, \forall(\mathbf{D},\mathbf{u})\in\overline{\mathbb{E}}_2$$

$$(11)-(15).$$

This model can be solved directly by commercial solvers such as GUROBI.

References

1. Shapiro, A. On duality theory of conic linear problems. In *Semi-infinite programming*, 135–165 (Springer, 2001).
2. Hanasusanto, G. A., Kuhn, D. & Wiesemann, W. A comment on "computational complexityof stochastic programming problems". *Math. Program.* **159**, 557—569 (2016).
3. Denton, B. T., Miller, A. J., Balasubramanian, H. J. & Huschka, T. R. Optimal allocation of surgery blocks to operating rooms under uncertainty. *Oper. Res.* **58**, 802–816 (2010).
